# Influence of Genetic Variations in Selenoprotein Genes on the Pattern of Gene Expression after Supplementation with Brazil Nuts

**DOI:** 10.3390/nu9070739

**Published:** 2017-07-11

**Authors:** Janaina L. S. Donadio, Marcelo M. Rogero, Simon Cockell, John Hesketh, Silvia M. F. Cozzolino

**Affiliations:** 1 Department of Food and Experimental Nutrition, Faculty of Pharmaceutical Sciences, University of São Paulo, São Paulo 05508-900, Brazil; smfcozzo@usp.br; 2 Department of Nutrition, Faculty of Public Health, University of São Paulo, São Paulo 01246-904, Brazil; mmrogero@usp.br; 3 Institute for Cell and Molecular Biosciences, Faculty of Medical Sciences, Newcastle University, Newcastle upon Tyne NE2 4HH, UK; simon.cockell@newcastle.ac.uk (S.C.); j.e.hesketh@newcastle.ac.uk (J.H.)

**Keywords:** SNPs, polymorphisms, microarray, transcriptomics, micronutrient

## Abstract

Selenium (Se) is an essential micronutrient for human health. Its beneficial effects are exerted by selenoproteins, which can be quantified in blood and used as molecular biomarkers of Se status. We hypothesize that the presence of genetic polymorphisms in selenoprotein genes may: (1) influence the gene expression of specific selenoproteins and (2) influence the pattern of global gene expression after Brazil nut supplementation. The study was conducted with 130 healthy volunteers in Sao Paulo, Brazil, who consumed one Brazil nut (300 μg/Se) a day for eight weeks. Gene expression of *GPX1* and *SELENOP* and genotyping were measured by real-time PCR using TaqMan Assays. Global gene expression was assessed by microarray using Illumina HumanHT-12 v4 BeadChips. Brazil nut supplementation significantly increased *GPX1* mRNA expression only in subjects with CC genotype at rs1050450 (*p* < 0.05). *SELENOP* mRNA expression was significantly higher in A-carriers at rs7579 either before or after supplementation (*p* < 0.05). Genotype for rs713041 in *GPX4* affected the pattern of blood cell global gene expression. Genetic variations in selenoprotein genes modulated both *GPX1* and *SELENOP* selenoprotein gene expression and global gene expression in response to Brazil nut supplementation.

## 1. Introduction

There is a considerable evidence to indicate that nuts are an important component of a healthy diet and this is thought to be at least partly due to their fatty acid composition and micronutrient content [1,2,3]. Brazil nuts (*Berthollethia excelsa*, family Lecythidaceae) are unique in also containing a high level of the micronutrient selenium (Se), and its use as a dietary supplement was able to increase the concentrations of biomarkers of Se status in different populations [4,5,6,7]. Se is an essential trace element that has an important role in human biology. There are 25 genes encoding selenoproteins with a wide range of functions, including antioxidant defense, redox function, thyroid hormone metabolism, immune function, reproduction and fertility [8,9]. Unlikely in most minerals that interact as cofactors in the active site of enzymes, Se is inserted as the amino acid selenocysteine (Sec) during translation. This process involves recoding the stop codon UGA to insert Sec and requires the presence of a stem-loop structure in the 3′untranslated region (3′UTR) of selenoprotein mRNAs (Sec Insertion Sequence or SECIS) and a specific tRNA for Sec (tRNA^[Ser]Sec^) [10].

Selenoprotein expression is regulated by Se supply, but different selenoproteins respond differently to available Se, depending on the specific tissue and the specific selenoprotein. As a result, there is a hierarchy in the response of selenoproteins to Se supply. For example, under Se-deficient conditions, Se is directed to the brain and endocrine tissues rather than to liver and kidneys [11]. Within the same tissue, some proteins have a preference for synthesis when the Se supply is limiting. This difference in regulation of selenoprotein expression reflects their physiological importance, and during deficiency states, the ones ranked high in the hierarchy have preference for synthesis [11].

The concept of hierarchy in the regulation of selenoprotein expression raises the possible use of molecular biomarkers of Se status in supplementation studies using humans and rodents. In mouse models, nine selenoprotein genes had reduced mRNA expression in the liver during Se-deficiency, including *GPX1*, *SELENOH*, *SELENOW*, *TXNRD1*, *TXNRD2*, *DIO1*, and *SELENOF*. This reduction ranked them low in the hierarchy and made them a possible target for use as molecular biomarkers in rodents [12]. Nevertheless, several human studies have failed to demonstrate an association of Se status and selenoproteins transcripts [13,14,15]. Only two studies have observed a positive relationship between Se supplementation and increased selenoprotein expression [16,17].

Genetic polymorphisms are an important source of inter-individual variation in response to nutritional supplementation [18]. Several single nucleotide polymorphisms (SNPs) in selenoproteins genes have been shown to be functionally significant and to affect the response of biomarkers of Se status to Se supplementation [19,20,21,22]. In particular, rs1050540 in *GPX1*, rs713041 in *GPX4* and rs7579 in the Selenoprotein P gene (*SELENOP*) are known to affect the expression of the respective selenoproteins. In the case of rs713041, the variant is a C > T substitution located in the 3’UTR of the *GPX4* gene and it affects Se incorporation in a cell culture model [23] and the response to Se supplementation in healthy adults [20]. It should be highlighted that rs713041 can modulate GPx4 activity by altering Sec insertion and protein binding to the 3′UTR [20]. rs7579 is a change G > A present in the 3′UTR of *SELENOP* and has been shown to affect Selenoprotein P (SePP) concentrations to Se supplementation in both European Americans and South Asians (12).

However, although Brazil nuts are a rich source of Se and therefore it is expected that Brazil nut supplementation would affect selenoprotein expression, no studies have investigated the influence of genetic polymorphisms on response to Brazil nut supplementation in healthy adults. In addition, none of the studies investigating the effect of either Se or Brazil nut Se supplementation in humans have considered the effect of genetic variants on the pattern of global gene expression. Therefore, the aims of the present study were twofold: firstly, to investigate the influence of three functional SNPs in selenoprotein genes (rs1050540, rs713041 and rs7579) on the expression of selenoproteins in response to Brazil nut supplementation, and secondly to use microarray analysis to assess the influence of rs713041, a well-characterized functional SNP in *GPX4*, on the pattern of global gene expression after Brazil nut supplementation in healthy adults.

## 2. Materials and Methods

### 2.1. Brazil Nut Supplementation and Blood Sampling

**Figure 1:**
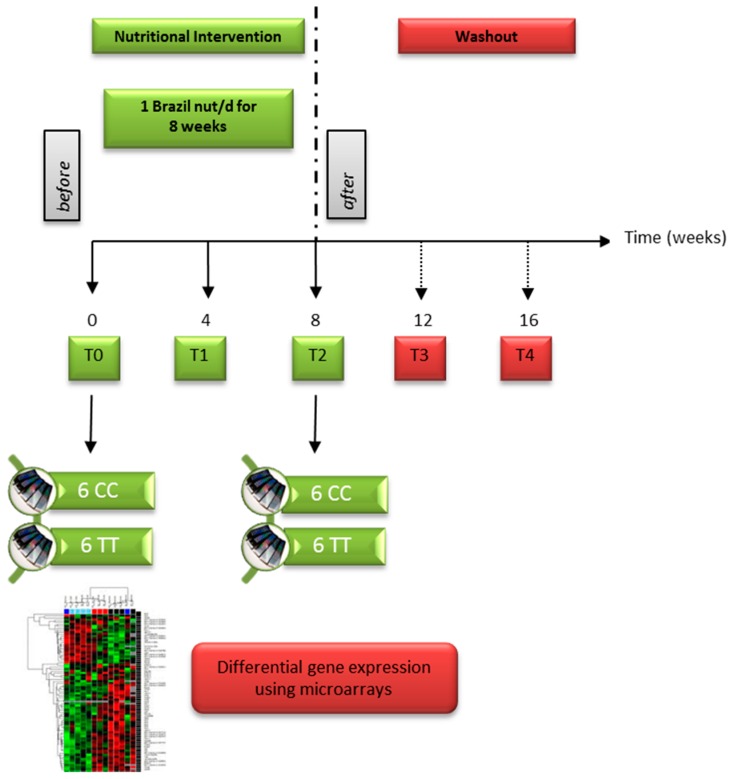
Intervention protocol of the Supplementation with Brazil Nuts study (SU.BRA.NUT) Biological sample collection for the microarray experiment is shown. CC indicates common genotype and TT indicates rare genotype for rs713041 in *GPX4* gene.

The present study involved 130 unrelated healthy volunteers with a mean age of 29.8 years old and a BMI of 23.3 kg/m^2^, who took part of the Supplementation with Brazil Nuts study (SU.BRA.NUT) described previously [24]. Volunteers taking multivitamins and mineral supplements, anti-inflammatory drugs, with excessive alcohol consumption, athletes, obese (BMI > 30) and with chronic diseases such as cancer, diabetes and cardiovascular disease were not included in the study. At the beginning of the study (baseline), 20 mL venous blood samples were drawn, and, subsequently, the volunteers took a daily supplement of one Brazil nut for eight weeks. At the end of four (4-week intervention) and eight weeks (8-week intervention) of supplementation, another 20 mL blood sample was taken, and then two more blood samples were taken after a further four (4-week washout) and eight weeks without intervention (8-week washout) (see Figure 1). Volunteers were asked to complete a control calendar and mark with an “x” when they consumed each nut throughout the intervention period. Written informed consent was signed by all volunteers before blood sampling. The protocol was approved by the Faculty of Pharmaceutical Sciences Ethical Committee (CAE: 00961112.3.0000.0067) and was conducted according to the Declaration of Helsinki. The study was registered at clinicaltrials.gov under the number NCT03111355.

### 2.2. Genotyping

Total genomic DNA was extracted from whole blood using a Purelink Genomic DNA Minikit (Invitrogen, Thermo Scientific, Carlsbad, CA, USA) and the final DNA concentration and purity were measured by spectrophotometry at 260 and 280 nm (NanoDrop ND 1000, Thermo Scientific, Wilmington, DE, USA). Genotyping was carried out by real-time PCR using the StepOne Plus Real Time system with Taqman SNP Genotyping Assays (Applied Biosystems, Thermo Scientific, Fostercity, CA, USA). The allelic discrimination was obtained by performing an endpoint read. The SNPs selected were located in the *GPX1* gene (rs1050450), the *GPX4* gene (rs713041), the *SELENOP* gene (rs3877899 and rs7579), the *SELENOS* gene (rs34713741) and the *SELENOF* gene (rs5845).

### 2.3. Selenoprotein Gene Expression

Total RNA was extracted from whole blood using a Ribopure Blood Kit (Ambion, Thermo Scientific, Austin, TX, USA) and final concentration and purity were measured spectrometrically in a NanoDrop ND 1000 spectrophotometer (NanoDrop ND 1000, Thermo Scientific, Wilmington, DE, USA). cDNA was synthesized by reverse trancriptase PCR using a High Capacity Reverse Transcriptase kit (Applied Biosystems, Thermo Scientific, Fostercity, CA, USA). Analysis of gene expression was performed by real-time quantitative PCR (qPCR) in the QuantStudio 12K Real-Time PCR System using Taqman Gene Expression Assays for *GPX1*, *SELENOP*, *SELENOS* and *SELENOF* (Applied Biosystems, Thermo Scientific, Fostercity, CA, USA). Glyceraldehyde phosphate dehydrogenase (GAPDH) mRNA was used as a reference gene. Relative gene expression was calculated based on the 2^−ΔΔCq^ method [25].

### 2.4. Microarray Analysis

Microarray analysis was carried out to investigate the influence of rs713041 in *GPX4* on the pattern of global gene expression after Brazil nut supplementation. Total RNA was extracted before and after nut supplementation from the whole blood of 12 volunteers previously genotyped (see Figure 1): 6 with the common genotype CC and 6 with the rare genotype TT for rs713041. Total RNA was extracted from whole blood using a Purelink Blood MiniKit (Ambion, Thermo Scientific, Austin, TX, USA). The integrity of these samples was checked by capillary electrophoresis using Tape Station 2000 (Agilent Technologies, Santa Clara, CA, USA) with the Agilent RNA Nano kit. Samples with a RNA integrity number (RIN) of above seven were used for whole genome microarray analysis by Service XS (Leiden, The Netherlands) using the Illumina HumanHT-12 v4 BeadChip (Illumina, San Diego, CA, USA). RNA quality control measurements were confirmed by Service XS using an Agilent Bioanalyzer (Agilent Technologies, Santa Clara, CA, USA), and then RNA labeling, amplification, and hybridization were performed. Raw microarray scan files were exported using the Illumina Beadstudio program and loaded into R for downstream analysis using the BioConductor and specific packages for each step of the bioinformatics analysis [26]. Probes with signals that fulfilled the criteria of the Illumina probe detection *p*-value of 0.05 were considered different. The bioinformatics analysis was performed by the Bioinformatics Support Unit, Faculty of Medical Sciences, Newcastle University, Newcastle upon Tyne, England, UK.

### 2.5. Gene Set Enrichment Analysis (GSEA)

The transcriptome data were analyzed by gene set enrichment analysis (GSEA), which ranks the genes in a list by their differential expression and tests for coordinated differences in a set of genes in response to a specific situation, rather than individual genes with increased or decreased expression in two conditions. One advantage of this integrated approach is the facility to interpret a large amount of data by identifying biological pathways and processes. In addition, GSEA considers the entire list of genes of the experiment, rather than only the ones that passed a fold-change cut-off. GSEA has been shown to be more sensitive than the traditional approach of single gene analyses [27]. The GSEA application from the Broad Institute, described previously [27], was used in the present work . Three files were created (dataset file.gct, phenotype file.cls and gene sets file.gmt) and loaded into the software. The dataset file contained the normalized microarray data, in our case with 19,835 probes and 23 arrays. The phenotype file contained the information about the experimental conditions, which were numbered. In this experiment, the genotypes for rs713041 and the supplementation were used. Therefore, four experimental conditions were created: 0 = CC_before, 1 = CC_after, 2 = TT_before and 3 = TT_after. The gene sets file was downloaded from the Molecular Signature Database v5.1 (MSigDB), an online collection of gene sets from different databases available for free to use with the GSEA application. The MSigDB has 8 different collections. Only two sets applicable to our context were used: C2, curated gene sets from online pathways databases and C5, Gene Ontology gene sets.

### 2.6. Statistical Analysis

Volunteers were selected for gene expression analysis based on their genotype that had been determined previously. For all statistical analysis, individuals who were homozygous and heterozygous for the rare alleles were combined together in one group, leaving the homozygous dominant in another category. Relative gene expression of each gene was normalized by the GAPDH reference gene using the 2^−ΔΔCq^ method. The final fold-change was used for statistical comparisons and submitted to normality tests using the Shapiro–Wilk test. The genotype effect before and after nuts was assessed by the Mann–Whitney test. The supplementation effect of each genotype was assessed by the Wilcoxon Test. Differences were considered significant if *p* < 0.05.

## 3. Results

The Supplementation with Brazil Nuts study (SU.BRA.NUT) was carried out to investigate the influence of genetic variations on the response to Brazil nut supplementation in biochemical and molecular biomarkers of Se status in healthy Brazilians. The study was conducted with 130 healthy adults, of which 66 were selected according to their genotype for analysis of gene expression of four selenoproteins and 12 were selected based on their genotype for rs713041 for microarray analysis. The mean ± standard deviation for Se content of the four batches used for the supplementation was 100.4 ± 5.3 μg/g. The average weight of the nuts was from 3 to 4 g, therefore each nut provided approximately 300 μg of Se, which is approximately five times higher than the Recommended Dietary Allowance (RDA) for adults of 55 μg/day.

### 3.1. Selenoprotein Gene Expression 

**Figure 2:**
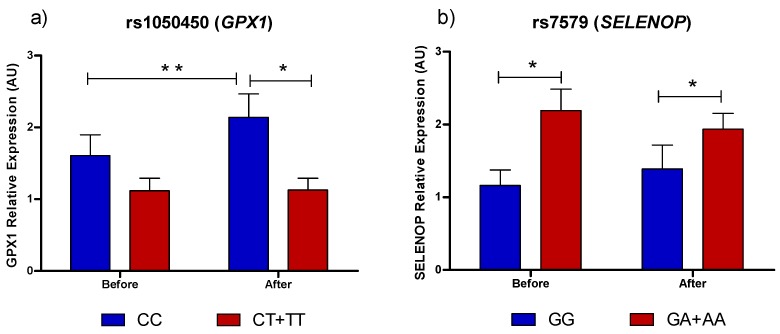
Selenoprotein expression in response to Brazil nut supplementation in previously genotyped volunteers. (**a**) GPX1 mRNA expression as a function of genotype for rs1050450; (**b**) SELENOP mRNA expression as a function of genotype for rs7579. * *p* < 0.05, Mann–Whitney test. ** *p* < 0.05, Wilcoxon test.

Gene expression of two selenoprotein genes (*GPX1* and *SELENOP*) is shown in Figure 2. *GPX1* mRNA expression was affected by genotype for rs1050450 with the increase in *GPX1* expression after supplementation observed only in CC individuals but not in CT or TT individuals (*p* = 0.026). After Brazil nut consumption, *GPX1* expression was lower in T-carriers compared to CC individuals (Figure 2a). rs7579 in *SELENOP* affected *SELENOP* expression before and after nut supplementation: *SELENOP* mRNA expression was higher in carriers of the rare allele A compared to GG individuals either before or after supplementation (Figure 2b, *p* < 0.05). No differences were observed for SELENOS and SELENOF mRNA expression in response to Brazil nut supplementation (results not shown).

### 3.2. Global Gene Expression

**Figure 3:**
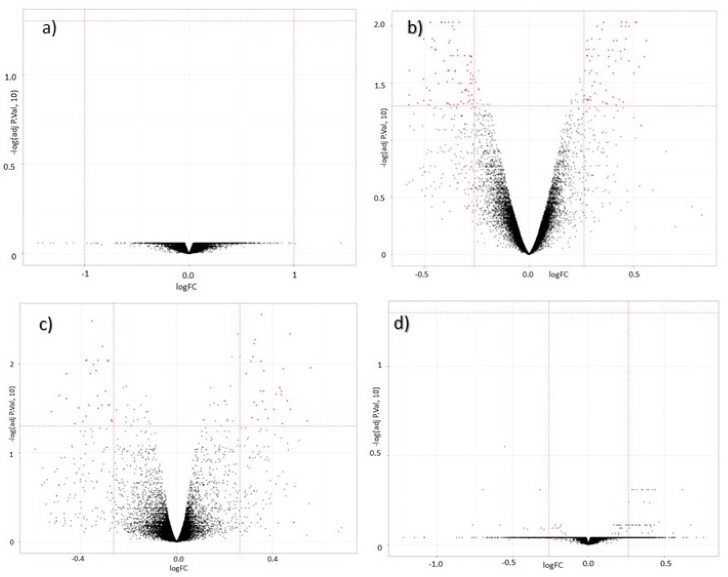
Volcano plots for the four experimental conditions investigated in the SU.BRA.NUT study. (**a**) Before supplementation comparing the genotypes CC × TT; (**b**) after supplementation comparing the genotypes CC × TT; (**c**) effect of the supplementation in the CC genotype and (**d**) effect of the supplementation in TT genotype.

**Figure 4:**
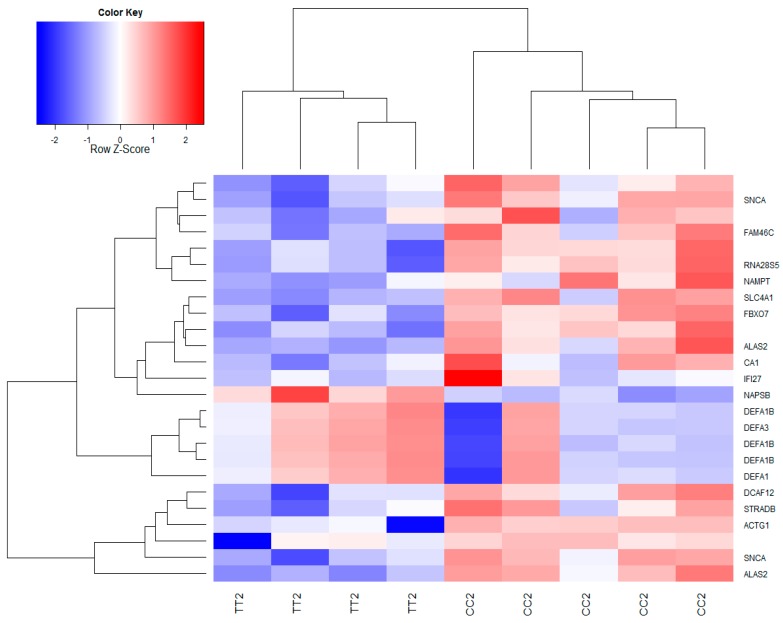
Heatmap showing patterns of differential expression in TT and CC genotype after Brazil nut supplementation. Red indicates genes with higher expression levels and blue genes with lower expression levels.

The overall pattern of differential gene expression before and after nut supplementation and as a function of genotype for rs713041 is shown in Figure 3. Before supplementation, as illustrated by Volcano plots, there was no effect of genotype on gene expression (Figure 3a). On the contrary, after supplementation, there was some evidence of genes differentially expressed comparing both genotypes, using a fold change of 1.0 and a *p*-value of 0.05 (Figure 3b). The pattern of differentially expressed genes before and after supplementation in CC and TT individuals is shown in Figure 3c,d. The effect of Brazil nut supplementation was significant only in individuals with the CC genotype (Figure 3c). No effect was observed for individuals with the less common TT genotype (Figure 3d). The heatmap in Figure 4 illustrates the gene expression pattern after supplementation comparing CC and TT individuals (referent to volcano plot 3b), and shows an opposite pattern of response of individuals with the different genotypes. Genes that were downregulated in TT individuals after nuts were upregulated in CC individuals.

Gene set enrichment analysis was carried out using 19,835 probes and 23 arrays. Both genotypes and the supplementation were used as conditions for the comparisons. The collection of gene sets available in the Molecular Signatures Database (MSigDB) as C2, curated gene sets from online pathways databases, and C5, Gene Ontology gene sets, were tested. No gene sets from C2 pathways were enriched either in CC individuals before and after nuts or in between CC and TT genotypes after nuts. However, 13 gene sets from the Cellular Component list from Gene Ontology (C5) related to ribosomes, Endoplasmatic Reticulum (ER) and Golgi compartments, and mitochondria were found to be enriched in TT individuals after nuts (Table 1). This effect of rs713041 in *GPX4* may reflect the importance of GPX4 in mitochondrial function [28]

## 4. Discussion

Previous works have investigated possible associations between Se supplementation and molecular biomarkers of Se status such as transcripts of selenoproteins in white blood cells in human studies [13,14,15,16]. These studies were not able to find an association between plasma Se biomarkers and selenoprotein expression after Se supplementation, except for one study conducted with healthy adults in the UK, which could observe the upregulation of some selenoprotein genes after Se supplementation [16]. The present study demonstrated that three genetic variants in selenoprotein genes (rs1050450, rs7579 and rs713041) affected the response to supplementation with Se-rich Brazil nuts at the transcriptional level. Furthermore, the results indicate that the SNP rs713041 in *GPX4* gene could modulate the pattern of global gene expression. This transcriptomic approach to investigating the response to Brazil nut supplementation based on the genetic profile has not been observed before.

The supplementation with one unit of Brazil nut in other populations significantly increased blood selenium levels [6,7], indicating that indeed the Brazil nuts are a rich source of dietary selenium. In our study, *GPX1* mRNA expression in whole blood was also increased by the nut supplementation. This result was different from three previous human studies that have investigated if Se supplementation affects selenoprotein transcript levels. A small study conducted in Denmark found no association of Se supplementation as Se-enriched milk, yeast or selenate for one week with *GPX1* mRNA expression [13]. Similarly, the five-year long PRECISE study and a longitudinal study conducted in the UK also found no association [14,15]. Nevertheless, two studies are in agreement with our results. One study conducted with healthy adults in the UK observed that the supplementation with 100 μg/day with sodium selenite for six weeks increased the expression of Selenoprotein K (*SELENOK*) and Selenoprotein 15 (*SELENOF*), showing that these selenoproteins are sensible to alterations of Se status [16]. A second study conducted with Alzheimer’s patients also found an increase in *GPX1* mRNA expression after supplementation with one unit of Brazil nuts for six months [17]. Possible explanations for this variation in response to Se supplementation could be either the presence of genetic variants, which most of the aforementioned works have not considered, the baseline Se status of the populations or the high level of Se provided by Brazil nuts. 

Interestingly, the increase in *GPX1* mRNA expression observed in our study was dependent on the presence of genetic polymorphisms. The increase in *GPX1* mRNA expression was significant only for individuals with the common CC genotype. No difference was observed in T-carriers. This genetic variation was associated with increased *GPX1* mRNA expression in other Brazilian work conducted with Alzheimer’s patients, but the authors found an increase in T-carriers instead of CC [17]. Although some studies have investigated the association of this SNP with differences in GPx1 activity [22,29,30], few studies have associated this variation with *GPX1* mRNA expression.

The presence of SNPs in *SELENOP* gene influenced its mRNA expression. The presence of the less common allele A for rs7579 was associated with an increase in *SELENOP* expression at baseline and after supplementation. Previous works with humans have not found an association between Se supplementation and *SELENOP* mRNA expression in white blood cells [13,15]. SePP is known to have two different isoforms in human plasma, the 60 kDa and the 50 kDa, and the presence of SNPs in *SELENOP* gene was associated with different proportions of the isoforms [31]. The 60 kDa isoform is more abundant in plasma and is found in higher proportion in the presence of the less common allele A for rs7579 [31]. One hypothesis was that the increase in *SELENOP* mRNA expression found only in A-carriers for rs7579 in our study could be related to the 60 kDa isoform being expressed more in plasma. Further studies are needed to confirm this association of rs7579 on *SELENOP* mRNA expression and the proportion of the 60 kDa in plasma.

Our work also had the goal of determining if genetic variants would influence the pattern of global gene expression in response to a natural source of Se, such as Brazil nuts. The SNP rs713041 in the *GPX4* gene was selected to test this hypothesis, as there is evidence that this SNP is functional [20,23,32]. It was observed that, although not statistically significant, the heatmap and volcano plots suggested the opposite response to Brazil nut supplementation based on genotype. To our knowledge, the association of rs713041 genotypes with the profile of global gene expression has not been observed previously. Moreover, GSEA showed that the biological processes and cellular compartments altered by the supplementation were related to protein synthesis, mitochondria and endoplasmatic reticulum. This supports the findings of previous humans studies that used microarrays to investigate the effect of Se supplementation [16,33]. These studies observed that processes related to protein biosynthesis were upregulated after Se supplementation. This could be explained by the molecular biosynthesis of selenoproteins, which needs the synthesis of a specific tRNA^[Ser]Sec^ for the amino acid selenocysteine, inserted in the proteins during translation. In addition, the effect of rs713041 in *GPX4* on mitochondrial pathways may reflect the importance of GPX4 in mitochondrial function [28]. One of the limitations of this study includes the small number of individuals to perform the analysis of the pattern of global gene expression, considering this a pilot study. Therefore, further work conducted with a higher sample size is needed to confirm our results.

## 5. Conclusions

In conclusion, the present study suggested that supplementation with Brazil nuts can modify the gene expression of some selenoproteins depending upon the presence of genetic polymorphisms. In addition, it has been demonstrated that the use of microarrays to investigate the pattern of global gene expression in response to a nutritional intervention with nuts is feasible, and that a genetic profile for a particular variant in *GPX4* (rs713041) possibly modulates global gene expression and is an important source of inter-individual variation. This could be relevant to direct future nutritional interventions for the use of molecular and biochemical biomarkers considering the interaction with the genetic variations.

## Figures and Tables

**Table 1:** Enriched gene sets from Gene Ontology (C5) in TT individuals for rs713041 in a GPX4 gene after supplementation with Brazil nuts compared with CC individuals. Gene sets were considered to be enriched at an (FDR) cut-off of 25%.

Name	Size	ES	NES	NOM *p*-Value	FDR *q*-Value
Cellular component					
Organellar ribosome	22	−0.69	−1.56	0.035	1.000
Mitochondrial ribosome	22	−0.69	−1.56	0.035	0.571
Early endosome	15	−0.74	−1.53	0.012	0.582
ER Golgi Intermediate compartment	20	−0.52	−1.52	0.004	0.496
Microtubule cytoskeleton	101	−0.41	−1.51	0.025	0.397
Ribosomal subunit	20	−0.66	−1.51	0.076	0.332
Intrinsic to endoplasmic reticulum membrane	23	−0.58	−1.50	0.035	0.320
Integral to endoplasmic reticulum membrane	23	−0.58	−1.50	0.035	0.280
Mitochondrial matrix	44	−0.55	−1.49	0.066	0.289
Mitochondrial lumen	44	−0.55	−1.49	0.066	0.260
Replication fork	16	−0.61	−1.48	0.014	0.242
Nuclear chromosome part	25	−0.55	−1.48	0.036	0.223
Golgi apparatus	166	−0.37	−1.45	0.008	0.280

ER: Endoplasmatic reticulum; ES: Enrichment Score; NES: Negative Enrichment Score; FDR: False Discovery Rate.
